# Public mental health during and after the SARS-CoV-2 pandemic: Opportunities for intervention *via* emotional self-efficacy and resilience

**DOI:** 10.3389/fpsyg.2023.1016337

**Published:** 2023-01-23

**Authors:** Melissa M. Karnaze, Brent M. Kious, Lindsay Z. Feuerman, Sarah Classen, Jill O. Robinson, Cinnamon S. Bloss, Amy L. McGuire

**Affiliations:** ^1^Herbert Wertheim School of Public Health and Human Longevity Science, University of California San Diego, San Diego, CA, United States; ^2^Department of Psychiatry, University of Utah, Salt Lake City, UT, United States; ^3^Center for Medical Ethics and Health Policy, Baylor College of Medicine, Houston, TX, United States

**Keywords:** public mental health, COVID-19, emotional self-efficacy, resilience, loneliness, depression, burnout

## Abstract

**Importance:**

During the pandemic, the number of United States adults reporting clinically significant symptoms of anxiety and depression sky-rocketed, up from 11% in 2020 to more than 40% in 2021. Our current mental healthcare system cannot adequately accommodate the current crisis; it is therefore important to identify opportunities for public mental health interventions.

**Objective:**

Assess whether modifiable emotional factors may offer a point of intervention for the mental health crisis.

**Design, setting, and participants:**

From January 13 to 15, 2022, adults living in the United States were recruited *via* Amazon Mechanical Turk to complete an anonymous survey.

**Main outcomes and measures:**

Linear regressions tested whether the primary outcomes during the SARS-CoV-2 pandemic (depressive and anxiety symptoms, burnout) were associated with hypothesized modifiable risk factors (loneliness and need for closure) and hypothesized modifiable protective factors (the ability to perceive emotions and connect with others emotionally; emotion-regulation efficacy; and resilience, or the ability to “bounce back” after negative events).

**Results:**

The sample included 1,323 adults (mean [SD] age 41.42 [12.52] years; 636 women [48%]), almost half of whom reported clinically significant depressive (29%) and/or anxiety (15%) symptoms. Approximately 90% of participants indicated feeling burned out at least once a year and nearly half of participants (45%) felt burned out once a week or more. In separate analyses, depressive symptoms (Model A), anxiety symptoms (Model B), and burnout (Model C) were statistically significantly associated with loneliness (βModel A, 0.38; 95% CI, 0.33–0.43; βModel B, 0.30; 95% CI, 0.26–0.36; βModel C, 0.34; 95% CI, 0.28–0.41), need for closure (βModel A, 0.09; 95% CI, 1.03–1.06; βModel B, 0.13; 95% CI, 0.97–0.17; βModel C, 0.11; 95% CI, 0.07–0.16), recent stressful life events (βModel A, 0.14; 95% CI, 0.10–0.17; βModel B, 0.14; 95% CI, 0.11–0.18; βModel C, 0.10; 95% CI, 0.06–0.15), and resilience (βModel A, −0.10; 95% CI, −0.15 to −0.05; βModel B, −0.18; 95% CI, −0.23 to −0.13; βModel C, −0.11; 95% CI, −0.17 to −0.05). In addition, depressive and anxiety symptoms were associated with emotional self-efficacy (βModel A, −0.17; 95% CI, −0.22 to −0.12; βModel B, −0.11; 95% CI, −0.17 to −0.06), and beliefs about the malleability of emotions (βModel A, −0.08; 95% CI, −0.12 to −0.03; βModel B, −0.09; 95% CI, −0.13 to −0.04). Associations between loneliness and symptoms were weaker among those with more emotional self-efficacy, more endorsement of emotion malleability beliefs, and greater resilience, in separate models. Analyses controlled for recent stressful life events, optimism, and social desirability.

**Conclusion and relevance:**

Public mental health interventions that teach resilience in response to negative events, emotional self-efficacy, and emotion-regulation efficacy may protect against the development of depressive symptoms, anxiety, and burnout, particularly in the context of a collective trauma. Emotional self-efficacy and regulation efficacy may mitigate the association between loneliness and mental health, but loneliness prevention research is also needed to address the current mental health crisis.

## Introduction

Chronic stress is associated with worse physical health and greater risk of suicide (Kennedy et al., 1988; van Heeringen and Mann, 2014; O’Connor et al., 2020), and there is evidence that it contributes to the development of psychiatric diagnoses, such as major depressive disorder (Hughes et al., 2017; Targum and Nemeroff, 2019; Cruz-Pereira et al., 2020). The widespread and prolonged stress related to the SARS-CoV-2 pandemic, and implicated social and economic strife, has been linked to several indices of worsened mental health: increased rates and severity of depression and anxiety (Ettman et al., 2020; Hossain et al., 2020; Racine et al., 2021), increased emergency room visits for mental health reasons (Holland et al., 2021; Krass et al., 2021), and, in some contexts, increased rates of substance abuse and lethal overdose (Mason et al., 2021; Panchal et al., 2021; Root et al., 2021). According to a Kaiser Family Foundation survey conducted in January 2021, 41.1% of adults in the United States reported clinically significant symptoms of anxiety or depression, elevated from 11.0% reporting symptoms in June 2019 (Panchal et al., 2021). Symptoms were more common in younger adults, with 56.2% of respondents ages 18–24 reporting symptoms of anxiety or depression, and 48.9% of respondents ages 25–49 reporting symptoms of anxiety or depression.

Some physicians are trained to screen for mental health symptoms, such as screening for suicide risk during emergency room visits (Roaten et al., 2021). The United States Preventive Services Task Force recommends depression screening for primary care patients (Calonge et al., 2009). However, rates of screening fall short of recommendations (Akincigil and Matthews, 2017), and among specific patient groups, randomized controlled trials of depression screening have had mixed results (Thombs et al., 2021). Using questionnaires rather than clinician interviews for screening can have unintended consequences such as overdiagnosis or misdiagnosis, especially when symptoms result from another health condition. Even with accurate screening, physician referral for mental healthcare may not result in treatment, as there are several barriers to treatment, such as mental health stigma (Chekroud et al., 2018). It is estimated, for instance, that in 2020, only 66% of adults with a major depressive episode received treatment (Major Depression, 2022). Large-scale efforts are needed to help address the rise of mental health symptoms in the general public. To maximize reach and reduce barriers to uptake, interventions may need to be delivered differently than the traditional, labor-intensive models in which individual, face-to-face care is provided directly by clinicians.

Pandemics are unique from other chronic stressors in at least three ways that have important implications for mental health. First, they are characterized by the need to maintain physical distance from others to prevent viral transmission, which can strain existing social relationships and reduce the social support they provide. This can contribute to feelings of loneliness, or the subjective experience of perceived social isolation. Second, the uncertainties associated with the course of the pandemic, government responses to it, and its economic impact could take a greater toll on those who are less tolerant of uncertainty and more prone to anxiety. Thus, another risk factor closely tied to the experience of living through a pandemic is a greater need for closure, or the tendency to want immediate solutions to problems and definitive answers to questions when they are not feasible. Third, pandemics can lead to cascading traumas, or stressful events that may exacerbate other stressful events, or make additional stressful experiences more likely to occur (Silver et al., 2021). Successive acute stressors, or chronic stressors are associated with worse health outcomes (Kennedy et al., 1988). In addition to structural factors related to stress exposure, stress responses, and support resources to cope with stress, there are several modifiable factors that can contribute to negative health effects of stress in the context of a pandemic, but these are modifiable and can thus be mitigated.

### Modifiable protective factors in the context of the pandemic

An important first step is to identify individual differences that are associated with mental health symptoms and can be modified through large-scale interventions. Given the need to social distance during a pandemic, behaviors that are conducive to social connection should be protective for mental health during a pandemic. An important factor in social relationships is having emotion-related skills conducive to social relationships, such as being able to perceive others’ emotions and empathize with others’ emotions. There are also several modifiable individual factors that attenuate negative health effects of stress. Well-documented psychological factors conducive to minimal perturbation or recovery to “baseline” after stress or adversity include high distress tolerance (Leyro et al., 2010; Arici-Ozcan et al., 2019; Felton et al., 2019), high self-esteem (Liu et al., 2014), secure attachment (Rasmussen et al., 2019), and high mental flexibility (Tol et al., 2013). These factors can be summarized by the construct of trait resilience, or self-reports of the tendency to “bounce back” after stressful events. Other well-documented protected psychological factors include low rumination and high cognitive reappraisal (Grierson et al., 2016), and low expressive suppression (Hu et al., 2014; Iacoviello and Charney, 2014). In addition to these specific emotion-regulation styles, researchers have studied the more general tendency to view one’s own emotional experiences as modifiable, which can reflect perceived ability to regulate or change one’s emotions (Kneeland et al., 2016). These three individual differences, emotional self-efficacy, resilience, and beliefs about the malleability of emotion, may be most amenable to short-term intervention and may be most easily scaled to address population-level healthcare needs, such as those produced by the pandemic.

### The present investigation

To identify modifiable protective factors associated with fewer mental health symptoms and less burnout during the SARS-COV-2 pandemic, we conducted a cross-sectional study of United States adults. We hypothesized that depression and anxiety symptoms would be associated with risk factors, namely greater loneliness and need for closure. We also hypothesized that when accounting for these risk factors, as well as other pandemic-related stressful life events, less depression and anxiety would be associated with multiple resilience factors, including more emotion-related skills conducive to sustaining close relationships, more emotion-regulation efficacy, and greater ability to “bounce back” after negative events (resilience). Other studies have shown each of these is associated with outcomes of interest, but to our knowledge, none has looked at all of these factors in conjunction. An analysis of the associations between multiple emotional risk and protective factors such as the one described here provides an opportunity to assess how different risk factors interact and assess which factors would have the greatest relative impact if modified.

## Method, design, and participants

United States adults (age 18 years and older) from the Amazon Mechanical Turk (MTurk; Shapiro et al., 2013; Thomas and Clifford, 2017) platform were eligible to participate in the study for $1 compensation if they had an MTurk approval rating of at least a 92%. MTurk participant pools have been reliably used to study psychological symptoms and interventions (Chandler and Shapiro, 2016). The cross-sectional survey was administered *via* online survey platform QualtricsXM from 13 January, 2022 to 15 January, 2022. Of the 1,745 complete survey responses, we excluded 237 with a completion time less than 5 min, and 174 with an incorrect response to any of the three attention check items per best practices to ensure high-quality responses (Peer et al., 2014; Smith et al., 2016) from MTurk participant pools. After demographic questions, the order of the remaining measures was randomized for each participant.

The Baylor College of Medicine Institutional Review Board approved the study (Protocol H-47626). Participants clicked “agree” to the study information page before completing the online survey.

### Measures

#### Outcomes

The 9-item Patient Health Questionnaire (Kroenke et al., 2001) assessed symptoms of depression and the 7-item Generalized Anxiety Disorder scale (Spitzer et al., 2006) assessed symptoms of anxiety. A single item measure of burnout from work was modified to assess feeling “burned out” (in general, or without referencing “work”), using a scale from 0 (Never) to 6 (Every day; Rohland et al., 2004).

#### Covariates

To account for the tendency to have a positive outlook and present oneself in a positive light in reports of mental health and burnout, we included an assessment of dispositional optimism versus pessimism using the Revised Life Orientation Test (Herzberg et al., 2006) and positive self-presentation bias as assessed by the short version of the Marlow-Crowne Social Desirability Scale (Fischer and Fick, 1993). Demographic information included: age, gender, race and ethnicity, relationship status, education, and personal financial impact of the SARS-COV-2 pandemic (see Table 1).

**Table 1:** Participant characteristics (*N* = 1,334).

Characteristic	Participants, No. (%)
Age, Mean (SD)	41.42 (12.52)
**Gender identity**	
Woman	636 (47.7)
Man	687 (51.5)
Non-binary	6 (0.4)
Prefer not to state	5 (0.4)
**Race and ethnicity**	
Asian	76 (6)
Black	100 (8)
Hispanic or Latino	71 (5)
White	962 (72)
Mixed/Other/Decline to state	125 (9)
Married/In a relationship	940 (70.5)
**Education**	
Less than college	368 (27.6)
College degree	727 (54.5)
More than college	239 (17.9)
**Financial impact from pandemic**	
Improved from pandemic	401 (30.1)
Not impacted by pandemic	501 (37.5)
Worsened by pandemic	432 (32.4)
Loneliness, Mean (SD)	18.12 (5.65)
Need for closure, Mean (SD)	63.02 (12.69)
Recent stressful events, Mean (SD)	110.13 (103.13)
Emotional self-efficacy, Mean (SD)	4.65 (1.27)
Beliefs about emotion malleability, Mean (SD)	3.46 (0.79)
Resilience, Mean (SD)	3.24 (0.84)
PHQ-9 depression, Mean (SD)	9.11 (7.60)
PHQ-9 severe depression cutoff (≥15)	392 (29.4%)
GAD-7 anxiety, Mean (SD)	7.55 (6.07)
GAD-7 severe anxiety cutoff (≥15)	200 (15%)
Burnout, Mean (SD)	3.07 (1.83)
Burnout reported at least weekly (≥4)	596 (45%)

#### Risk factors

We also assessed three factors hypothesized as risks for mental health symptoms and burnout. First, we included an assessment of stressful life events experienced within the past year, using The Holmes-Rahe Life Stress Inventory (Holmes and Rahe, 1967), which included 43 life events that are typically regarded as positive (e.g., marriage) or negative (e.g., death of a spouse). Second, we included two psychological factors. The Need for Closure Scale (Webster and Kruglanski, 1994; Pierro and Kruglanski, 2008; Roets and Van Hiel, 2011) measured a latent construct including items assessing tendencies to: prefer predictability, order, and structure; feel discomfort with ambiguity; and be decisive and close-minded. The short-form UCLA Loneliness Scale (ULS-8) was used to assess the frequency with which participants experienced subjective feelings of loneliness (Hays and DiMatteo, 1987).

#### Modifiable protective factors

We included three psychological factors hypothesized to play a modifiable protective role in mental health outcomes and burnout. The Personal Beliefs about the Malleability of Emotions (De Castella et al., 2013) measure assessed the degree to which participants perceived having the ability to change or control their emotions. The Brief Resilience Scale (Smith et al., 2008) assessed the tendency to “bounce back” or recover from negative-emotion-eliciting events or experiences. Emotional self-efficacy, or emotion-related skills conducive to wellbeing and social relationships, was assessed using the “emotionality” factor of the short-form of the Trait Emotional Intelligence measure and included two items representing abilities to: (1) perceive emotions; (2) express emotions; (3) empathize with others’ emotions, and (4) sustain close relationships with others (Petrides et al., 2007; Petrides, 2009).

### Statistical approach

We used separate hierarchical linear regression analyses in IBM SPSS Statistics 27 (IBM, 2020) to test whether hypothesized risk and modifiable protective factors were associated with the outcomes of depressive symptoms, anxiety symptoms, and burnout. We first entered demographic variables (age, sex, whether in a relationship, race and ethnicity, and education) and other covariates (social desirability and optimism) at Step 1 of each of the three models. We then entered hypothesized risk factors (recent stressful life events, need for closure, and loneliness) and hypothesized modifiable protective factors (emotional self-efficacy, emotion malleability beliefs, and resilience) at Step 2 to examine their association with depressive symptoms (Model A), anxiety symptoms (Model B), and burnout (Model C) beyond any effects of demographics and other covariates. Next, we assessed the three modifiable protective factors as potential modifiers of associations between loneliness and worse outcomes in separate models. In a fully adjusted Step 3, we entered in the multiplicative interaction term for emotional self efficacy*loneliness. Then, separately, we tested whether the association between loneliness and outcomes was a function of each of the other two modifiable protective factors (beliefs about emotion malleability*loneliness, and then resilience*loneliness).

## Results

**Figure 1:**
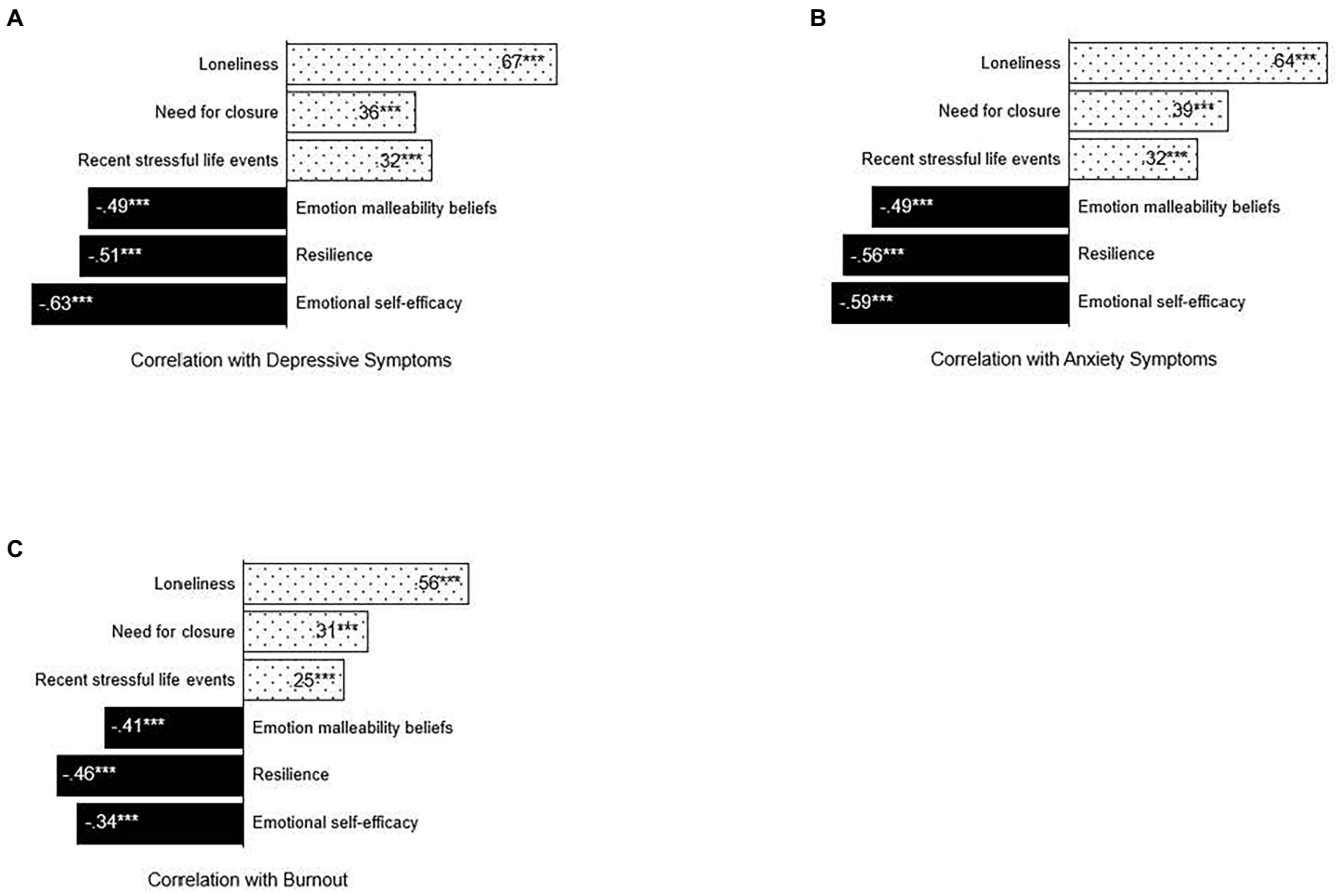
Correlations between modifiable protective/risk factors and mental health symptoms and burnout. Zero-order correlations between modifiable protective (solid black)/risk factors (dotted) and **(A)** depressive symptoms, **(B)** anxiety symptoms, and **(C)** burnout. All correlations are greater than zero at *p* < 0.001. *N* = 1,334.

A total of 1,334 United States adults participated in the survey (636 women, 687 men, 6 non-binary, and 5 prefer not to state; mean (SD) age in years, 41 (12.52); 72% white; 71% in a relationship). Participant characteristics are displayed in Table 1. Descriptive statistics and zero-order correlations for the three risk and three modifiable emotional factors are displayed in Supplementary Table 1, and zero-order correlations between modifiable protective/risk factors and the outcome variables are displayed in Figure 1.

Consistent with reports of elevated depressive and anxiety symptoms after the start of the pandemic, nearly a third of participants (29.4%; *N* = 389) reported clinically significant depressive symptoms warranting further assessment by a clinician (PHQ-9 score of 15 or higher), while approximately 17% (*N* = 227) reported depressive symptoms in the moderate range (PHQ-9 score between 10 and 14). Similarly, approximately 15% (*N* = 200) reported clinically significant anxiety symptoms (GAD-7 score of 15 or greater), while approximately 27% (*N* = 361) reported moderate anxiety symptoms (GAD-7 score between 10 and 14). Approximately 90% (*N* = 1,209) of participants indicated feeling burned out at least once a year and nearly half of participants (*N* = 596; 45%) felt burned out once a week or more. Depressive and anxiety symptoms were strongly statistically significantly correlated, *r* = 0.90, *p* < 0.001. Burnout was also correlated with both depressive (*r* = 0.66, *p* < 0.001) and anxiety symptoms (*r* = 0.67, *p* < 0.001).

### Risk and modifiable protective factors and associations with mental health outcomes and burnout

As shown in Table 2, risk factors were associated with more, and protective factors with fewer/lower, mental health symptoms and burnout frequency. For ease of interpretation of both risk and modifiable protective factors, we summarize the results for each risk and then each modifiable protective factor in the three separate regression models predicting the three outcomes (depressive symptoms, anxiety symptoms, and frequency of burnout).

**Table 2:** Standardized regression coefficients for modifiable protective and risk factors in relation to mental health symptoms and burnout.

Predictor variable	Outcome variable					
	Depressive symptoms		Anxiety symptoms		Burnout	
Modifiable protective factors	*β* (95% CI)	*P*	*β* (95% CI)	*P*	*β* (95% CI)	*P*
Emotion malleability beliefs	−0.08 (−0.12, −0.03)	0.001	−0.09 (−0.13, −0.04)	<0.001	−0.01 (−0.06, 0.05)	0.83
Resilience	−0.10 (−0.15, −0.05)	<0.001	−0.18 (−0.23, −0.13)	<0.001	−0.11 (−0.17, −0.05)	<0.001
Emotional self-efficacy	−0.17 (−0.22, −0.12)	<0.001	−0.11 (−0.17, −0.06)	<0.001	0.03 (−0.03, 0.09)	0.34
**Risk factors**						
Recent stressful events	0.14 (0.10, 0.17)	<0.001	0.14 (0.11, 0.18)	<0.001	0.10 (0.06, 0.15)	<0.001
Need for closure	0.09 (0.05, 0.12)	<0.001	0.13 (0.10, 0.17)	<0.001	0.11 (0.07, 0.16)	<0.001
Loneliness	0.38 (0.33, 0.43)	<0.001	0.31 (0.26, 0.36)	<0.001	0.34 (0.28, 0.41)	<0.001

Standardized beta coefficients (*β*) are presented for all models. Models included the following covariates: binary gender, sex, race, and ethnicity, whether in a relationship, better or worse off financially due to the pandemic (reference group: no impact of pandemic), less than or more than four-year college degree attainment (reference group: four-year college degree), social desirability, and dispositional optimism. Analyses only included participants with binary gender identity due to too small of a sample size for non-binary gender, which was underpowered to test for any statistically significant differences for this group. *N* = 1,323.

#### Risk factors

##### Loneliness

In three separate models predicting each outcome, each standard deviation increment in loneliness was associated with more depressive symptoms, (*β* = 0.38, *p* < 0.001), more anxiety symptoms, (*β* = 0.31, *p* < 0.001), and more burnout, (*β* = 0.34, *p* < 0.001).

##### Need for closure

In the same models, each standard deviation increment in need for closure was associated with more depressive symptoms (*β* = 0.09, *p* < 0.001), more anxiety symptoms (*β* = 0.13, *p* < 0.001), and more frequent burnout (*β* = 0.11, *p* < 0.001).

##### Stressful life events

Similarly, each standard deviation increment in stressful life events was associated with more depressive symptoms (*β* = 0.14, *p* < 0.001), more anxiety symptoms (*β* = 0.14, *p* < 0.001), and more frequent burnout (*β* = 0.10, *p* < 0.001).

#### Modifiable protective factors

##### Emotional self-efficacy

In three separate models predicting each outcome, each standard deviation increment in emotional self-efficacy was associated with fewer depressive symptoms, (*β* = −0.17, *p* < 0.001), and fewer anxiety symptoms, (*β* = −0.11, *p* < 0.001), but was not associated with frequency of burnout (*p* = 0.34).

##### Beliefs about malleability of emotions

Similarly, each standard deviation increment in beliefs about emotion malleability was associated with fewer depressive symptoms (*β* = −0.08, *p* = 0.001) and fewer anxiety symptoms (*β* = −0.09, *p* < 0.001), but was not associated with frequency of burnout (*p* = 0.83).

##### Resilience

Each standard deviation increment in resilience was associated with fewer depressive symptoms (*β* = −0.10, *p* < 0.001), fewer anxiety symptoms (*β* = −0.18, *p* < 0.001), and less frequent burnout (*β* = −0.11, *p* < 0.001).

### Exploratory analyses of modifiable protective factors that mitigate the effects of loneliness

In models predicting all three outcomes, the effect size for loneliness was larger (*β*s > 0.30) than effect sizes for the other risk factors as well as the modifiable protective factors that were statistically significantly associated with outcomes (0.08 > *β*s > 0.19). We thus explored whether the modifiable protective factors would mitigate the statistical effects of loneliness on outcomes. Because there were no main effects of emotional self-efficacy or beliefs about malleability of emotions predicting burnout, we did not test for whether those factors moderated the effects of loneliness on burnout.

#### Emotional self-efficacy

**Figure 2:**
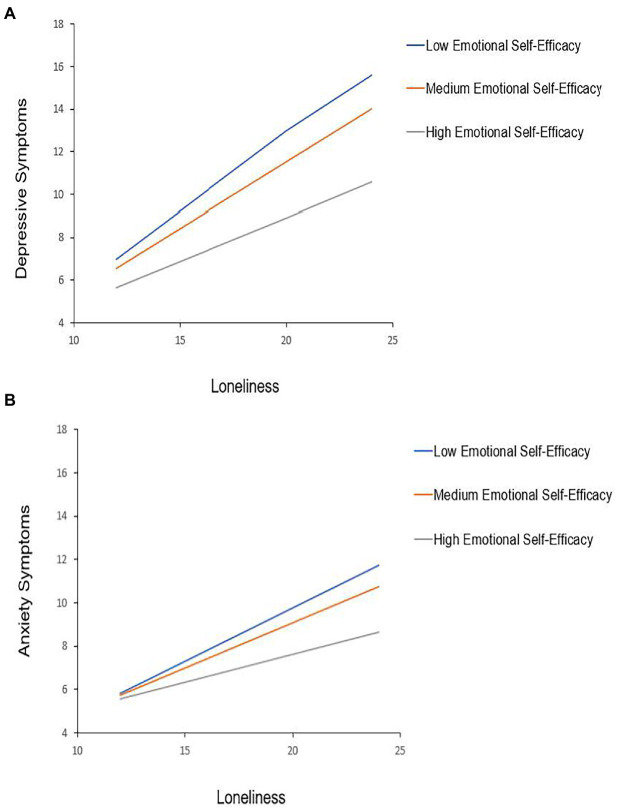
Emotional self-efficacy moderates the association between loneliness and mental health symptoms. The association between loneliness (possible score range: 8–32) and mental health symptoms [**(A)** depression possible score range: 0–27; **(B)** anxiety possible score range: 0–12] is moderated by emotional self-efficacy in separate hierarchical regression models with all specified covariates included. The simple slopes for 16th (3.38), 50th (4.13), and 84th (5.75) percentiles of emotional self-efficacy scores are displayed (all *p* < 0.001). Models included the following covariates: binary gender, sex, race, ethnicity, whether in a relationship, better or worse off financially due to the pandemic (reference group: no impact of pandemic), less than or more than 4-year college degree attainment (reference group: 4-year college degree), social desirability, and dispositional optimism. Analyses only included participants with binary gender identity because a sample size for non-binary gender meant the study was underpowered to test for any statistically significant differences for this groups. *N* = 1,323.

Individuals with greater emotional self-efficacy showed weaker associations between loneliness and depressive symptoms (*p*-interaction <0.001) and weaker associations between loneliness and anxiety symptoms in a separate model (*p*-interaction <0.001; see Figure 2).

#### Beliefs about malleability of emotions

In a model only testing for the interaction between beliefs about emotion malleability and loneliness, individuals with greater endorsement of beliefs about emotion malleability showed weaker associations between loneliness and depressive symptoms (*p*-interaction <0.001) and weaker associations between loneliness and anxiety symptoms in a separate model (*p*-interaction <0.001).

#### Resilience

Individuals with greater resilience showed weaker associations between loneliness and depressive symptoms (*p*-interaction <0.001) and weaker associations between loneliness and anxiety symptoms in a separate model (*p*-interaction <0.001). There was no interaction between resilience and loneliness in predicting burnout (*p* = 0.94).

## Discussion

In January 2022, we assessed the prevalence of self-reported symptoms of anxiety, depression, and burnout among 1,323 United States adults and examined whether three hypothesized modifiable protective factors were associated with fewer depressive and anxiety symptoms and less frequent burnout. As expected, we found that the more participants perceived having the ability to understand and use their emotions to create and maintain positive social connections (emotional self-efficacy), and the more they perceived having the ability to regulate their emotions in general (beliefs about the malleability of emotion), the fewer symptoms of depression and anxiety they reported. In addition, the more participants reported the tendency to recover or bounce back from negative life experiences (resilience), the fewer depressive and anxiety symptoms they reported, and the less frequently they felt burned out. Taken together, these findings suggest that believing that one is capable of using their emotional experiences to feel socially connected to others in close and positive ways, and feeling capable of self-regulating emotional experiences in general, as well as in the aftermath of negative life events, played a protective role for mental health after nearly 2 years since the start of the SARS-CoV-2 pandemic. Moreover, greater endorsement of the belief that one tends to recover from negative emotions resulting from stressful events also played a protective role in the degree of burnout.

Interventions designed to promote these three factors, either individually or in combination, may be protective against the development or severity of depressive and anxiety symptoms, particularly in the context of a collective trauma. Existing school interventions can inform efforts to increase emotional self-efficacy. For instance, the RULER approach, a scalable elementary school program based on emotional intelligence (Brackett et al., 2019), which conceptually encompasses emotional self-efficacy, led to social and emotional learning among children. Importantly, feeling able to recognize and understand emotions may be more important than accuracy (Barrett and Gross, 2001) when it comes to approaching others and having meaningful social interactions. Likewise, feeling close and connected to others may matter more than the actual degree of social “contact” as was demonstrated during the pandemic, when physical distancing was encouraged and in some places mandated for public health (Cacioppo et al., 2010; Cohen-Mansfield et al., 2016; Domènech-Abella et al., 2021; Gubler et al., 2021). Research on mindsets also supports the idea that beliefs about the malleability of emotion can protect against anxiety and depressive symptoms. For example, encouraging the mindset that personality is malleable among high schoolers and decreased the incidence of clinically significant depressive symptoms 9 months later (Miu and Yeager, 2015). Among community college students, encouraging the mindset of stress as adaptive decreased math evaluation anxiety and improved math exam performance (Jamieson et al., 2016). Similarly, experiments designed to promote mindsets of stress and physiological arousal as helpful rather than harmful have positive effects on performance (Crum et al., 2020) and health (Jamieson et al., 2013). A cost-effective and relatively brief online intervention taught the growth mindset that intelligence can be developed to high school students across the United States (Yeager et al., 2019). However, this intervention was only effective at improving students’ grades when teachers were supportive of such a view (Yeager et al., 2022). Following the conceptual model proposed by Yeager et al. (2019), we posit that interventions to increase emotional self-efficacy and change beliefs might have beneficial effects when people have specific emotion-regulation or relationship goals as well as a supportive environment to work toward their goals. Another note of caution is that there may be unique challenges for teaching or instilling emotional self-efficacy among populations with more neurodiversity or diagnoses of personality disorders characterized by difficulties in empathy or emotion regulation.

In terms of emotion-regulation efficacy, work has shown that it is possible to increase the use of an adaptive emotion-regulation strategy that promotes wellbeing (Gross and John, 2003), cognitive reappraisal of an emotion-eliciting event (Denny and Ochsner, 2014). Experiments have also increased perceived emotion-regulation efficacy (Kneeland et al., 2016), which should have positive implications for psychological distress and subjective wellbeing (De Castella et al., 2013). In addition, encouraging functional beliefs about specific emotions (Gutentag et al., 2022), or emotions in general (Ford and Gross, 2019) may provide a starting point to intervene on emotion regulation. However, more naturalistic research is needed to determine how to modify perceptions of resilience in response to negative events, in part because resilience can be conceptualized as spanning the months or years after a stressful life event (Troy et al., 2023). Hardiness is a construct related to resilience that encompasses commitment to personal development, perceived control, and approaching challenge, and it has been associated with indices of better mental health symptoms and attenuated distress responses to acute laboratory stressors (Taylor et al., 2013; Matthews et al., 2019). Hardiness training has improved academic performance (Maddi, 2002; Maddi et al., 2002, 2009). Another construct related to resilience is grit, and interventions to increase grit have also been successful (Credé et al., 2017; Park et al., 2020).

Importantly, our analyses accounted for covariates that could have been related to both modifiable protective factors and mental health and burnout, such as financial impacts from the pandemic, the tendency to have an optimistic versus pessimistic outlook, and positive self-presentation bias. Analyses also accounted for three important risk factors, subjective feelings of loneliness, the need for cognitive closure or simple solutions and definitive answers to uncertainty, and stressful life events experienced within the past year. As expected, the higher participants scored on each of these risk factors, the more depressive and anxiety symptoms, and more frequent feelings of burnout, they tended to report. Findings suggest that stressful life events within the past year played a role in mental health symptoms and burnout. Moreover, less ability to adapt to the chronic stressors and accept the uncertainty inherent to a pandemic, or greater need to have cognitive closure, was associated with worse outcomes. Our findings suggest that in contrast, divergent thinking, or considering alternate ways of interpreting events and reality-testing the results of any particular response to a problem or situation, is likely to be a protective factor. Prior to the pandemic, divergent thinking was associated with fewer depressive symptoms (Liknaitzky et al., 2018) and increasing divergent thinking in the context of the pandemic decreased anxiety symptoms (Zuo et al., 2021). Also, recent work has shown that mindsets about the pandemic had a self-fulfilling pattern of quality of life 6 months later, such that stronger endorsement of a mindset that “the pandemic can be an opportunity” was protective (Zion et al., 2022). Efforts to increase divergent thinking, and tolerance of ambiguity, could also benefit from the cognitive behavioral and constructivist therapeutic approaches (Neimeyer, 2009; Hofmann et al., 2012) which have explicit goals of challenging undesirable perceptions of events to train increasingly functional ways of viewing the world.

It may seem logical for health professionals to screen for these risk factors along with a battery of other mental health assessments to identify clinically significant symptoms. However, this approach faces intrinsic limitations, such as lack of adequately trained staff, low rates of completed referrals, and a risk of overdiagnosis. Moreover, routine screening is intrinsically aimed at detecting more severe symptoms of anxiety, depression, and related conditions. Population-wide interventions could usefully augment a screening-based approach, as they could be delivered to persons with symptoms that fall below clinical thresholds. Similarly, they promise to provide primary prevention, as early stress inoculation is protective against the negative effects of stressful events later in life (Seery et al., 2010). These population-wide interventions could be delivered through schools or places of employment. A recent meta-analysis showing that there have been increases in loneliness during the pandemic (even though loneliness was a problem internationally before the pandemic) suggests that it may be incumbent upon institutions to contribute to interventions to mitigate loneliness (Ernst et al., 2022). In support of this approach, a meta-analysis of burnout interventions found that organization-led interventions were associated with stronger treatment effects relative to physician-directed interventions (Panagioti et al., 2017).

Loneliness showed the largest effect size of all of the hypothesized risk and modifiable protective factors, suggesting that irrespective of interventions designed to increase modifiable protective factors or promote coping with stressful life events, loneliness prevention research is needed to address the mental health crisis. Unfortunately, to our knowledge, there are few evidence-based interventions designed to prevent or reduce loneliness that are applicable at a population level (Masi et al., 2011; Lim et al., 2019). Accordingly, the current study suggests the importance of developing interventions that target loneliness. Scalable, brief interventions have been used to increase positive mindsets about stress (Crum et al., 2020) and social belonging (Walton and Cohen, 2011) and such designs could promote mindsets protective against loneliness, such as those that are conducive to a sense of “oneliness” (Jeste et al., 2020) and connection with others. Previous efforts have mostly aimed at increasing social skills and developing new social relationships; however, loneliness is distinct from social isolation/support and may require adjustments to emotional self-efficacy and regulation that improve perceptions of social connection, which may or may not reflect actual social connectedness.

Given the effect of loneliness in predicting the outcome variables, in follow-up analyses, we sought to investigate whether the most salient modifiable protective factor we assessed, emotional self-efficacy, would moderate the association between the loneliness and mental health outcomes. In exploratory models, we tested the interaction between emotional self-efficacy and loneliness in predicting depressive and anxiety symptoms. We found that among participants with more emotional self-efficacy, the association between loneliness and more depressive symptoms, and between loneliness and more anxiety symptoms, were statistically significantly weaker. These findings suggest that the perceived ability to recognize and understand emotions in oneself and others, as well as to utilize personal emotions to enhance positive social connections (e.g., through empathy) may mitigate the negative effects of loneliness on mental health during a collective trauma that required physical distancing and isolation in order to prevent viral transmission of COVID-19. However, it will be important to test whether increasing emotional self-efficacy can offset the hypothesized negative impact of loneliness on mental health symptom onset or severity during a collective trauma. It will also be important to determine whether individual constructs within emotional self-efficacy each serve a protective role, or if all constructs in combination are protective.

The study had several limitations. First, because of its cross-sectional design, we were unable to assess longitudinal changes in depression, anxiety, and burnout over the course of the pandemic, limiting inferences about the effects of stress related to COVID-19. Second, although MTurk samples have been reported to be more diverse than convenience samples (Buhrmester et al., 2016), they have also been shown to be somewhat younger and more educated than the United States general population (Paolacci et al., 2010). While participants in this sample were similar to the United States population in terms of average age (41.4 years old vs. 38.8 years old; Bureau, 2022b) and gender distribution (Population Distribution by Sex, 2022), they reported higher educational attainment (72% college degree or higher vs. 37.9%) and 72% self-reported as White compared to 62% White from the 2020 United States Census (Bureau, 2022a). It should be noted that the prevalence of clinically significant depressive symptoms in our sample (29%) was similar to that found (23%) in a national sample surveyed using a shorter (2-item) version of the scales by the National Center for Health Statistics and the Census Bureau in January 2022 in response to the pandemic (CDC, 2021). However, future research is needed to determine whether the modifiable factors we examined serve a protective role against depression, anxiety, and burnout in samples across various socioeconomic backgrounds and ethnic and racial identity. Although we utilized best practices including attention checks and screening for high approval ratings when using MTurk participant pools to improve the validity of our responses (Peer et al., 2014), there is no way to guarantee the legitimacy of MTurk survey responses. Future research should explore these psychological outcomes and potentially mediating factors with a nationally representative sample. Third, due to the cross-sectional design, the study was unable to capture variations in stress and associated symptoms that may have corresponded to variations in the severity of the pandemic; data were collected during the wave of infections associated with the Omicron variant. Likewise, any unique effects of the SARS-CoV-2 pandemic on participants, such as personal health effects or effects on family members or relationships, were not assessed. While the inventory of stressful life events did include several types of traumatic experiences that could have directly resulted from the pandemic (e.g., divorce, death of a family member or friend, personal illness or illness of a family member, and job loss), the overall measure assigned stress scores to both negative and positive life events. Thus, future research should include measures of perceived stress as well as validated measures of specific stressors (such as illness of self or family, financial strain, or stressors related to childcare) which have disproportionately affected individuals of minoritized groups or backgrounds of lower socioeconomic status (SES) than those within our sample (Broadbent et al., 2020). The study was also unable to include other SES factors potentially more directly related to the psychological effects of the pandemic, such as income, spiritual belief, and political affiliation. Finally, because the study relied on self-report, we were limited to participants’ perceived emotional abilities rather than their actual abilities.

In conclusion, public mental health interventions teaching resilience in response to negative events, emotional self-efficacy, and emotion-regulation efficacy may be protective against the development or severity of depressive and anxiety symptoms, particularly in the context of a collective trauma. Emotional self-efficacy may mitigate the negative effects of loneliness on mental health, but loneliness prevention research is also needed to address the mental health crisis.

## Data availability statement

The raw data supporting the conclusions of this article will be made available by the authors, without undue reservation.

## Ethics statement

The Baylor College of Medicine Institutional Review Board approved the study (Protocol H-47626). Written informed consent for participation was not required for this study in accordance with the national legislation and the institutional requirements.

## Author contributions

MK, BK, LF, JR, CB, and AM: conceptualization. MK, BK, LF, SC, JR, CB, and AM: methodology and writing—review and editing. MK and CB: formal analysis. SC and JR: data curation. MK and BK: writing—original draft. All authors contributed to the article and approved the submitted version.

## Funding

This work was supported, in part, by the Center for Empathy and Technology within the T. Denny Sanford Institute for Empathy and Compassion at the University of California San Diego.

## Conflict of interest

The authors declare that the research was conducted in the absence of any commercial or financial relationships that could be construed as a potential conflict of interest.

## Publisher’s note

All claims expressed in this article are solely those of the authors and do not necessarily represent those of their affiliated organizations, or those of the publisher, the editors and the reviewers. Any product that may be evaluated in this article, or claim that may be made by its manufacturer, is not guaranteed or endorsed by the publisher.
