# Supplementary material for: Public mental health during and after the SARS-CoV-2 pandemic: Opportunities for intervention via emotional self-efficacy and resilience
Source: Front Psychol. 2023 Jan 23;14:1016337. doi: 10.3389/fpsyg.2023.1016337 (PMC9899813; doi:10.3389/fpsyg.2023.1016337)
Supplement: Supplementary file 1 [file Table_1.pdf]

Supplementary Table 1. Descriptive Statistics and Zero-Order Correlations among Risk and Modifiable Protective Factors for Depressive and Anxiety Symptoms

				Correlations					
Variable	M	SD	Range	1	2	3	4	5	6
1. Loneliness	18.12	5.65	8.00-32.00	1	.30***	.18***	-.62***	-.47***	-.56***
2. Need for closure	63.09	12.69	15.00-90.00		1	.12***	-.33***	-.22***	-.28***
3. Recent stressful events	110.13	103.13	0.00-801.00			1	-.14***	-.09***	-.12***
4. Emotional self-efficacy	4.65	1.13	1.50-7.00				1	.56***	.48***
5. Beliefs about emotion malleability	3.46	0.79	1.00-5.00					1	.53***
6. Resilience	3.24	0.84	1.00-5.00						1

Note. *** $p < .001$. $N = 1334$.
